# Stent Thrombosis and Restenosis with Contemporary Drug-Eluting Stents: Predictors and Current Evidence

**DOI:** 10.3390/jcm12031238

**Published:** 2023-02-03

**Authors:** Francesco Condello, Carmen Spaccarotella, Sabato Sorrentino, Ciro Indolfi, Giulio G. Stefanini, Alberto Polimeni

**Affiliations:** 1 Department of Biomedical Sciences, Humanitas University, 20090 Milan, Italy; 2 IRCCS Humanitas Research Hospital, 20089 Milan, Italy; 3 Division of Cardiology, Department of Advanced Biomedical Science, Federico II University, 80138 Naples, Italy; 4 Division of Cardiology, Department of Medical and Surgical Sciences, Magna Graecia University, 88100 Catanzaro, Italy; 5 Mediterranea Cardiocentro, 88122 Naples, Italy; 6 Department of Pharmacy, Health and Nutritional Sciences, University of Calabria, 87036 Rende, Italy

**Keywords:** drug-eluting stent, stent thrombosis, restenosis, DES, PCI

## Abstract

Iterations in stent technologies, advances in pharmacotherapy, and awareness of the implications of implantation techniques have markedly reduced the risk of stent failure, both in the form of stent thrombosis (ST) and in-stent restenosis (ISR). However, given the number of percutaneous coronary interventions (PCI) performed worldwide every year, ST and ISR, albeit occurring at a fairly low rate, represent a public health problem even with contemporary DES platforms. The understanding of mechanisms and risk factors for these two PCI complications has been of fundamental importance for the parallel evolution of stent technologies. Risk factors associated with ST and ISR are usually divided into patient-, lesion-, device- and procedure-related. A number of studies have shown how certain risk factors are related to early (1 month) versus late/very late ST (between 1 month and 1 year and >1 year, respectively). However, more research is required to conclusively show the role of time-dependence of risk factors also in the incidence of ISR (early [1 year] or late [>1 year]). A thorough risk assessment is required due to the complex etiology of ST and ISR. The most effective strategy to treat ST and ISR is still to prevent them; hence, it is crucial to identify patient-, lesion-, device- and procedure-related predictors.

## 1. Introduction

Implantation of drug-eluting stents (DES) is a consolidated therapy for the treatment of both stable and unstable coronary artery disease (CAD). The first DES implantation dates back to 1999 when Eduardo Sousa in Brazil implanted the first sirolimus-eluting stent, which became available as Cypher stent (Cordis Corp, Miami, FL, USA) in 2002. At that time, the need to find an alternative to bare metal stents (BMS) arose from the high rates of in-stent restenosis (ISR) and target lesion revascularization (TLR) associated with this technology. Uncontrolled vascular smooth muscle cells (VSMC) proliferation and intimal migration, together with extracellular matrix production after BMS deployment, caused ISR in up to 30% of patients. In an effort to minimize the aberrant reaction caused by BMS, several drugs targeting thrombosis, platelet activation, inflammation, and VSMC proliferation have been tested. Coating BMS with anti-proliferative drugs such as sirolimus or paclitaxel led to a significant reduction in the risk of ISR as compared to BMS [1,2,3,4]. First-generation DES, such as the Cypher sirolimus-eluting stent and Taxus paclitaxel-eluting stent (Boston Scientific, Natick, MA, USA), undoubtedly represented a breakthrough in stent technology. However, over time, early-generation DES were associated with a small but steady risk of stent thrombosis (ST), which appeared more than a year after stent implantation. Awareness of molecular and pathological mechanisms of vessel healing and the understanding of the rheological modifications induced by stent implantation guided over the years several iterations in one of the three main components of DES, and the advent of contemporary DES allowed reaching an excellent efficacy and safety profile. Although the introduction of increasingly effective technologies and pharmacological advances have led to a significant reduction in stent failure, ST and ISR still occur, albeit at a fairly low rate. However, since the number of percutaneous coronary interventions (PCI) performed annually worldwide is increasing and the complexity of treated patients is growing, the two aforementioned PCI complications represent a relevant public health issue.

The aim of this review is to summarize contemporary DES technologies, the main risk factors associated with ST and ISR, and how advances in our understanding of these two complications have paved the way for the design of new, increasingly effective, and safe devices.

## 2. Evolution of Drug-Eluting Stents

DES are essentially based on three main components: a metallic stent backbone, an anti-proliferative agent, and a drug carrier (usually a polymer coating) [5,6]. The main differences in contemporary DES, as compared to early generation DES, may focus on one or more of the three components.

- Stent platform: contemporary DES are made to shorten the time it takes for the stented artery segment to heal, which is what caused first-generation DES to have a higher risk of very late ST than BMS [7,8]. The first-generation DES were built on a platform of stainless steel (iron, nickel, and chromium) with struts that were 130–150 μm thick. Cobalt chromium (CoCr) and platinum chromium (PtCr), two different metallic alloys, were used in newer-generation DES to achieve thinner stent struts (<100 μm), reduce strut-related changes in shear stress, and enable faster and thorough endothelial strut coverage while maintaining an adequate radial strength [9,10,11]. Additionally, the number of connectors and crowns has been shrinking in newer-generation stents, and contemporary DES have 2–3 connectors and 6–7 crowns with improved deliverability, flexibility, and conformability without any trade-off in radial and longitudinal strength [12].
- Polymer coating on the stent surface acts as a drug carrier and enables effective and controlled drug release at the arterial stented site. However, once the anti-proliferative medication has entirely been released, the polymeric material is no longer needed. Over time, the persistence of polymer coating may cause inflammatory responses within the arterial wall, impairing the stented artery’s ability to heal [13]. To overcome these issues, contemporary DES contain more biocompatible durable fluorinated or biodegradable polymers (made of lactic or glycolic acids which fully resorb by hydrolysis after the completion of drug release). A further iteration has been developed with polymer-free DES (PF-DES), which release the anti-proliferative drug directly from the stent surface without the need for a polymeric carrier.
- The anti-proliferative drug released from the polymer or stent surface prevents VSMC proliferation, minimizing the growth of neointimal tissue inside the stent. On first-generation DES platforms, paclitaxel and sirolimus were introduced. By binding to the tubulin component of microtubules, paclitaxel suppresses their detachment from centrosomes, blocking the cell cycle. Sirolimus instead inhibits the mammalian target of rapamycin (mTOR), which prevents the advancement of the cell cycle, cell migration, and protein synthesis. It has repeatedly been demonstrated that sirolimus-eluting DES have a stronger anti-restenotic efficacy than paclitaxel-eluting DES [14]. This might be because sirolimus has a larger therapeutic index and distinct tissue kinetics than other drugs. Accordingly, the -limus family of drugs (which differ from each other in structure, molecular weight, potency, and lipophilicity) are used in newer-generation DES.

### 2.1. Durable-Polymer Drug-Eluting Stents

Durable-polymer DES (DP-DES), also known as second-generation DES, saw three main innovations as compared to first-generation DES: (1) the use of the new metallic alloys CoCr and PtCr instead of stainless steel in the stent backbone; (2) the introduction of new durable polymer coatings to reduce the inflammatory response, platelet activation, and fasten vessel re-endothelization; (3) the use of new -limus drugs.

The Endeavor zotarolimus-eluting stent (ZES) (Medtronic, Inc., Minneapolis, MN, USA) contains a polymer similar to the phospholipid phosphorylcholine molecules found in cellular membranes. Up to 95% of the anti-proliferative drug is released within the first two weeks after implantation. The lack of anti-proliferative drug following this brief period led to unexpectedly high rates of restenosis and repeat revascularization [15]. The Endeavor ZES was updated to contain BioLinxTM, a novel polymer with a hydrophilic surface that repels thrombogenic plasma proteins, to improve drug release kinetics and assure efficient control of neointimal tissue formation. About 85% of the drug is released after 2 months thanks to the new polymer, and the remaining 15% is released within 6 months (Table 1).

The Xience (Abott Vascular, Inc., Santa Clara, CA, USA) and the Promus Element (Boston Scientific, Natick, MA, USA) everolimus-eluting stents (EES) contain a double layered polymer coating: the first layer is a base coat made of poly n-butyl methacrylate (PBMA), the second layer is a co-polymer of vinylidene fluoride and hexafluoropropylene (PVDF-HFP), which reduces platelet activation and adhesion and prolongs the release of everolimus (25% during the first day and 75% during the first month). When CoCr and PtCr EES were compared in patients with one or two de novo lesions in the PLATINUM Trial, comparable rates of target lesion failure (TLF) and low ST incidence at 1-year follow-up were observed [16].

### 2.2. Biodegradable Polymer Drug-Eluting Stents

The poly-D,L-lactide acid (PDLLA), poly-L-lactide acid (PLLA), or poly-lactic-co-glycolic acid (PLGA) generally used to coat biodegradable polymer DES (BP-DES) are converted into water and carbon dioxide molecules after the anti-proliferative drug is eluted, leaving only a metallic platform within the stented vessel. Stent backbones consist of PtCr, CoCr, and stainless steel. A highly lipophilic sirolimus derivative known as Biolimus was embedded as a novel member of the -limus family. Even if there are many BP-DES which are very similar to one another, some significant differences still exist [17] (Table 2). New coating technologies were also developed in BP-DES. To prevent coating failure at the specific spot, which could eventually lead to major inflammatory injury, Terumo Interventional Systems designed a gradient style of coating in which the portions that underwent the greatest physical stress were left uncovered. To completely prevent the risk of cracking, only the central section of the struts was covered. To minimize the inhibition of endothelial cell proliferation and promote more physiological vessel healing, some manufacturers introduced the thin coating strategy and coated only the abluminal surface of the stent. Some additional features are typical of each stent, such as the amorphous silicon carbide layer on the luminal surface of the Orsiro stent (Biotronik AG, Buelach, Switzerland), the circumferential coating of anti-CD34 antibodies on the COMBO stent (OrbusNeich Medical, Fort Lauderdale, FTL, USA) to enhance endothelization, and the Micropore Technology on the Yukon Choice PC stent (Translumina GmbH, Hechingen, Germany).

### 2.3. Polymer-Free Drug-Eluting Stents

By altering the stent surface and drug-matrix formulations, PF-DES enable a controlled release of the anti-proliferative drug from the stent surface without the requirement of a polymer. Three PF-DES that have been developed so far have gained large-scale clinical trial experience and the CE mark for use in Europe (Table 3). Numerous techniques to alter or cover the surface of the stent have been reported. These either physically or chemically support drug loading.Direct coating, crystalline coating, nano- or microporous surface coating, inorganic porous coating, reservoir-based coating, nanoparticle coating on the stent, and self-assembled monolayer coating on stent surfaces are examples of coating strategies used in PF-DES [18].

## 3. Bioresorbable Scaffolds

Current generation DES present some residual drawbacks, likely related to permanent metallic platforms and durable polymers, including persistent local inflammation, incomplete endothelial coverage, late or very-late ST, impaired vessel remodeling, restricted vasomotion, and limited options for future target vessel revascularization [19]. The main feature of bioresorbable scaffolds (BRS) is the use of bioresorbable materials, which can be completely eliminated from the body by excretion and assimilation after complete cleavage into small molecules. The main difficulty in producing these devices is striking a balance between bioresorption and acceptable mechanical properties. The optimal platform needs significant break strains to be able to survive deformations from the crimped to expanded states, high elastic moduli to impart radial strength, and low yield strains to lessen the amount of recoil and overinflation required to accomplish a target deployment. The bioresorbable backbone of modern BRS can be characterized as either polymeric (consisting of a bioresorbable polymer) or metallic (comprised of a bioresorbable metal alloy) (Table 4). However, current generation BRS failed to challenge standard therapies, showing inferior outcomes to conventional DES and posing concerns regarding safety [20,21,22,23,24,25]. Future refinements in newer-generation BRS with optimized implantation strategies and proper intravascular imaging may shed new light on the field.

## 4. Risk Factors of Stent Thrombosis and In-Stent Restenosis with Contemporary DES

ST and ISR are key mechanisms of stent failure requiring repeat revascularization [26,27,28].

### 4.1. Stent Thrombosis

**Figure 1:**
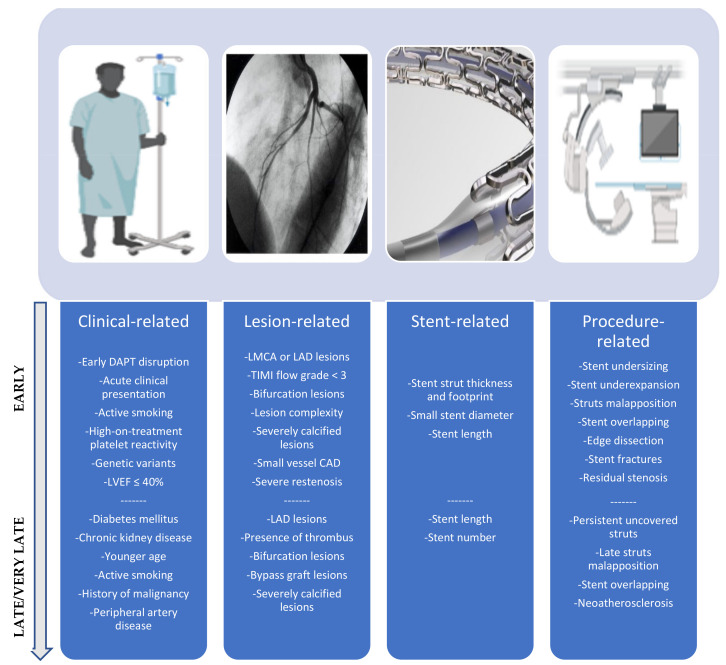
Overview of the main factors associated with stent thrombosis.

ST is a rare but fearsome complication of PCI. The evolution of PCI techniques, innovations in stent technologies, and advancements in antithrombotic therapy has significantly reduced the incidence of this complication [28,29,30,31,32]. ST is usually classified as early (within 30 days after stent implantation, further differentiated into acute—within 24 h of PCI and subacute—from 24 h to 30 days), late (between 1 month and 1 year after implantation), or very late (>1 year after implantation). Based on the degree of diagnostic certainty, ST is further classified as definite, probable, and possible [33]. Early ST is a devastating event associated with high mortality and morbidity rates as compared to late and very late ST. A meta-analysis reported 10% vs. 4% in-hospital mortality and 25% vs. 14% 1-year mortality for early and late/very late ST, respectively [34]. Several risk factors have been associated with the occurrence of this severe PCI complication, and over the years, a significant improvement in understanding mechanisms of ST has been made. Risk factors associated with this complication are usually divided into patient-, lesion-, device- and procedure-related (Figure 1).

It is noteworthy that the strength of the link between a risk factor and ST changes over time [35]. In the KoST (Korea Stent Thrombosis) registry enrolling 123 Korean patients receiving DES and presenting with ST, low left ventricular ejection fraction (LVEF), acute clinical presentation, lower stent diameter, and DES ISR were independent predictors of both early and late ST, bifurcation interventions were associated with early ST, while younger age, chronic kidney disease, and left anterior descending artery (LAD) lesion PCI emerged as correlates of late ST [36]. In a pooled analysis of 3 randomized trials and 4 registries that included 11,219 patients undergoing PCI with CoCr EES, dual antiplatelet therapy (DAPT) interruption before 30 days after stent implantation resulted as a strong predictor of early ST [37]. In the Dutch Stent Thrombosis Registry, which includes 437 patients treated with both BMS and DES who experienced ST, early clopidogrel disruption after the index PCI was associated with ST, mainly with early events. Stent under-sizing, uncovered dissection, sub-optimal procedural outcome, presence of intermediate CAD proximal and distal to the target lesion, history of malignancy, lack of aspirin use, LVEF ≤ 30%, bifurcation lesions, any DES, and stent number were among the predictors of early ST. Glycoprotein IIb/IIIa inhibitors acted as preventive agents against the development of early ST. Stent undersizing, cancer history, intermediate CAD close to the target lesion, peripheral artery disease, diabetes mellitus, bifurcation lesions, total stent length, and younger age were all independently linked to the development of late ST [38]. In a single-center observational study that included 1019 patients undergoing PCI with both BMS and DES, high-on-treatment platelet reactivity was associated with early but not late ST [39]. In the prospective case-control DESERT (International Drug-Eluting Stent Event Registry of Thrombosis) study, which included 492 cases of late/very late ST, younger age, African American ethnicity, active smoking at the time of DES implantation, multivessel disease, overlapping stenting, total stent length, saphenous vein graft lesion (SVG) location, LAD PCI, the presence of thrombus, and final in-stent diameter stenosis were the most important clinical and angiographic correlates of late/very late ST [40]. In the REAL-ST (Retrospective Multicenter Registry of ST After First- and Second-Generation DES Implantation) registry enrolling 313 patients treated with second-generation DES and presenting with definite ST, acute clinical presentation, LVEF < 40%, current smoking, prior PCI, severely calcified lesions, left main coronary artery lesions, stent overlap, and residual stenosis > 20% were associated with early ST; younger age, hemodialysis, ST-elevation myocardial infarction (STEMI) at presentation, LVEF < 40%, severely calcified lesions, and ISR resulted as correlates of late ST, while LAD PCI and ISR were predictors of very late ST [41]. Additionally, some variants in genes involved in clopidogrel and lipids metabolism have been associated with an increased risk of ST, with a strong association with subacute ST [42,43].

All three components of DES can have an impact on the risk of ST. Strut thickness and width, between-struts distance, struts number, polymer coating, and drug eluted can affect blood flow dynamics and several components of the vessel healing response, including inflammatory response, platelet adhesion, VSMC, and endothelial cell proliferation. Thicker struts, a short distance between struts, and an increased stent footprint (expressed as the ratio between strut surface and vessel surface) may increase local shear stress, cause platelet activation, and prevent re-endothelization [35,44,45]. In a meta-analysis that included 77 studies and 99,039 patients treated with 10 different types of DES, the ultrathin Orsiro BP-DES was associated with a significantly lower rate of ST as compared with Nobori/Biomatrix and Resolute DES [46]. Long-term persistence of polymer coating after anti-proliferative drug elution may trigger an inflammatory and/or hypersensitivity response, and causes the drug to be eluted inhomogeneously due to the presence of surface roughness where platelets can adhere. To date, data about the superiority of newer polymer coating technologies to reduce ST are lacking [47], but Optical Coherence Tomography (OCT) studies showed a significantly lower percentage of persistent uncovered struts with BP-DES [48,49] and PF-DES [50] as compared with DP-DES, albeit this finding was not confirmed in other trials [51]. A meta-regression analysis of 49 randomized trials reported a significant reduction of early but not late and very late ST using thinner struts DES and a decreased incidence of very late ST using newer-generation DES, reflecting the importance of rheological alterations (mainly depending on strut thickness) induced by stent implantation in the first phase and the relevance of complete struts coverage (mainly depending on the polymer biocompatibility), at a later stage, when antithrombotic therapy has already been remodulated with the discontinuation of DAPT [52].

The major pre- and post-operative parameters related to ST have also been clarified by intravascular imaging studies. One of the main procedure-related risk factors for ST is stent underexpansion. A minimal in-stent area (MSA) to reference vessel area ratio of 0.8 has been linked to ST and primarily correlates with early events. MSA lower than 4.5 mm^2^ or 5.5 mm^2^ using intravascular ultrasound (IVUS) or OCT, respectively, has also been linked to ST [53,54,55]. Additionally, stent oversizing >10%, which involves expanding the stent to a diameter that is 10% bigger than the reference vessel diameter to provide a smaller footprint, was associated with an eightfold lower incidence of ST compared to minimal oversizing in a pooled analysis of 14 trials on DES. It should be noted that this effect was significantly less prominent in vessels over 2.75 mm, whose footprint is already small [56]. Stent struts malapposition, which is defined as a lack of contact between the struts’ abluminal surface and the vessel wall, can begin soon after stent implantation as a result of the use of a stent that is too small for the target vessel, because of localized vessel enlargement or vessel asymmetry, or as a result of an asymmetrical distribution in the case of calcified lesions [53,54,55]. In spite of this, malapposition can develop years after implantation due to positive vascular remodeling, which is most likely caused by inflammatory and/or hypersensitive reactions that cause the vessel to expand outward and subsequently lose contact with the stent struts. An Expert Consensus Document of the European Association of Percutaneous Cardiovascular Interventions (EAPCI) considers an axial distance <0.4 mm and a longitudinal length < 1 mm of malapposed stent struts as optimization targets because below the aforementioned thresholds, full neointimal integration is expected at follow-up [57]. Uncovered struts were adjudicated as the main mechanism of early ST in the PRESTIGE (Prevention of Late Stent Thrombosis by an Interdisciplinary Global European Effort) study [54]. Early after stent implantation, neointimal tissue production and re-endothelization process are in progress, so it is expected to have a high percentage of uncovered stent struts. In this phase, it is likely that other factors, along with the pro-thrombotic non-endothelized stent struts, play a more important role in the thrombotic abrupt vessel closure. While the persistence of isolated uncovered struts years after DES implantation, when DAPT has already been discontinued and stent struts should be completely endothelized, may significantly increase the risk of late and very late ST. In a Korean study that included 489 patients undergoing PCI with DES implantation and subsequent OCT follow-up between 6–18 months, the post-intervention minimal lumen diameter and a percentage of uncovered struts ≥ 5.9% resulted as independent predictors of major safety events, including cardiovascular death, myocardial infarction, and ST [58]. Edge dissection has been associated with a high rate of ST and adverse cardiovascular events [59]. Based on the results of the CLI-OPCI (Centro per la lotta contro l’infarto-Optimization of PCI) and HORIZON-AMI (Harmonizing Outcomes with Revascularization and Stents in Acute Myocardial Infarction) studies, edge dissection disrupting the vessel media, extended >60°, and >2 mm long are considered those with the highest risk of ST [60,61]. Geographical lesion miss emerged as a further predictor of ST in several studies [38,60]. An MLA > 4.5 mm^2^, either at distal or proximal stent edges, is the threshold considered significant for PCI optimization [57]. Severe restenosis, modifying blood flow dynamics, increases the risk of late ST. In the PRESTIGE trial, OCT revealed that up to 19% of patients with late ST had significant restenosis. Furthermore, IVUS evidence of a link between ST and an inhomogeneous, immature in-stent neointima suggests that both ST and ISR may represent unfavorable phenomena based on similar biological processes [62]. Tissue prolapse after stent implantation, defined as tissue extrusion from inside the stent area, may be due either to lesion protrusion or, in the context of acute clinical presentation, to the protrusion of athero-thrombotic material and has been identified as an OCT predictor of early ST. A most important causal role is attributed to tissue prolapse in the context of acute coronary syndromes as compared to chronic coronary syndromes [57,61,63]. Stent fractures, defined as the complete or partial separation of a stent that was contiguous after the original stent implantation, stimulate the peri-strut inflammatory response and modify blood flow laminarity and have been associated with both ISR and ST [64,65] Neoatherosclerosis has been found to be the most important OCT predictor of very late ST [53,54,55]; vascular injury at the time of stent implantation and delayed vascular healing associated with abnormal neointimal growth, fibrin deposition, and inflammatory response are the key mechanisms implied in this phenomenon [66].

### 4.2. In-Stent Restenosis

Despite advancements in DES technology, ISR and the need for repeat revascularization continue to be the most common reasons for stent failure, occurring at a rate of 1–2% per year with modern DES platforms [67,68]. Every year about 10% of all PCI procedures are performed to treat ISR, but reported rates have reached as high as 20% in some studies [69,70,71]. The accepted definition of ISR is a diameter stenosis >50% within the stented segment (i.e., the stent and a 5 mm border proximal or distal to the stent) [27,68]. The occurrence of ISR may significantly impact long-term clinical outcomes after PCI. Indeed, recent data from the National Cardiovascular Data Registry (NCDR) CathPCI records compared outcomes among 653,304 patients, of which 10.2% undergoing ISR-PCI and 89.8% of de-novo lesion PCI, the former were associated with a higher incidence of major adverse cardiovascular and cerebrovascular events (MACCE) at 3 years, including higher incidence of all-cause mortality, myocardial infarction, repeat revascularization, TLR, and stroke [72]. About 25% of patients with ISR present an acute clinical presentation, more often with unstable angina, and less often with myocardial infarction compared to matched control undergoing de novo lesion PCI [73]. Despite first- and second-generation DES having been associated with a 60% relative risk reduction of ISR compared to BMS, the rate of ISR recurrence remains high and increases exponentially with the number of re-interventions performed [69,70]. Furthermore, as follow-up lengthens (i.e., 5–10 years), a late catch-up phenomenon is seen in the rates of ISR between DES and BMS [74].

Vessel injury after stent implantation triggers VSMC proliferation and extracellular matrix production, resulting in neointima formation. During vascular remodeling, as after stent implantation, VSMC undergo a phenotype switch from a contractile to synthetic and proliferative status. The subsequent accumulation of dedifferentiated VSMC in the intima is crucial in vessel healing and ISR. Phenotype switching and VSMC migration are regulated by several genetic and non-genetic mechanisms [75,76,77]. Neoatherosclerosis is characterized by rapid (over months to years) accumulation of lipid-laden foamy macrophages within the neointima, with or without degenerative calcifications and necrotic core formation [66]. New iterations in stent technologies led over the years to substantial changes in ISR pathomorphosis. The quantitative and qualitative representation of the three main components of ISR (VSMC, extracellular matrix, and neoatherosclerosis) has changed since newer-generation DES ISR is often hypocellular and proteoglycan-rich and VSMC usually express a contractile or intermediate phenotype, while in BMS-ISR synthetic VSMC play a major role along with a moderate proteoglycan content. Neoatherosclerosis is accelerated with first-generation DES, rare with BMS, and can develop over the long term with newer-generation DES [78,79,80]. The two types of stents have quite different time courses for neointimal formation. Late lumen loss with BMS often reaches a peak 6 to 8 months after implantation and then gradually declines. Contrarily, through 5 years following implantation, there is a slow and progressive neointimal buildup with DES. While DES-ISR is more commonly linked to focal or edge-related patterns, BMS-ISR is more frequently linked to diffuse patterns [27]. Intravascular OCT morphological findings also differed between early and late (>1 year) second-generation DES ISR. Early ISR is more often associated with stent under expansion and neointimal hyperplasia, while neoatherosclerosis prevails in late ISR [81]. This has been confirmed in a recent study in which 512 patients were evaluated by OCT for ISR of second-generation DES. Overall, neoatherosclerosis was the prevalent mechanism of ISR, with incidence ranging from 20% at 1 to 3 years and reaching above 70% at 7 years [82].

**Figure 2:**
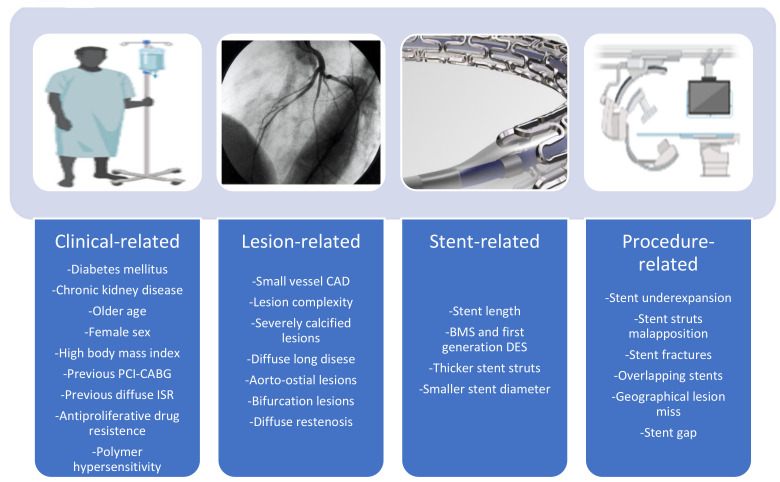
Overview of the main risk factors of in-stent restenosis.

As with ST, for ISR, several risk factors have been identified, and these are usually classified as patient-, lesion-, stent-, and procedure-related (Figure 2). Although some studies have identified different risk factors for early (<1 year) versus late (>1 year) ISR, further studies are needed to definitively demonstrate the role of time dependence also in ISR.

Diabetes mellitus, chronic kidney disease, advanced age, female sex, and higher body mass index are clinical predictors of ISR. Additionally, drug resistance, genetically determined or secondary to drug exposure, such as hypersensitivity and inflammatory responses triggered by the metallic stent backbone, DES polymer, and anti-proliferative released drug, can promote the formation of neointima [83]. In a retrospective clinical study that included 246 patients with multivessel disease undergoing PCI with second-generation DES, older age, current smoking, and advanced chronic kidney disease were independent predictors of ISR [84]. A retrospective study including 394 patients undergoing PCI with second-generation DES showed that the independent predictors of late ISR differed from those of early ISR. Specifically, previous PCI, diabetes mellitus, and postprocedure residual stenosis resulted as independent predictors of early ISR, while previous PCI and C-reactive protein (CRP) levels were correlates of late ISR [85]. However, other studies failed to show an association between baseline CRP and restenosis [86]. The presence of diabetes mellitus and a history of bypass surgery were associated with restenosis independently of the stent implanted in a large European registry [71]. In most studies conducted thus far, the constant clinical predictor of restenosis has been found to be diabetes mellitus. Patients with diabetes mellitus represent a special subgroup with more extensive CAD, complex lesions, and a high prevalence of dose-dependent resistance to anti-proliferative mTOR inhibitors [87,88]. Recently, the SUGAR (Second-generation drUg-elutinG Stents in diabetes) trial, which included 1175 diabetic patients undergoing PCI and randomized to Cre8 EVO or Resolute Onyx stents, showed that the Amphillimus PF-DES was non-inferior to Resolute Onyx with regard to target lesion failure and even superior to Resolute Onyx in a prespecified superiority analysis [89]. Moreover, a trend toward lower rates of clinically-indicated TLR was observed. Also, evidence is emerging about the association between autoimmune disorders and the risk of ISR [90].

Lesion factors associated with ISR include vessel type, size, and lesion characteristics. In a retrospective study enrolling patients previously treated for ACS, ISR was more prevalent in the LAD artery, followed by the left circumflex, right coronary artery, and finally, the left main coronary artery [91]. In a cohort of 10,004 patients with 6–8-month angiographic surveillance, small vessel size and complex lesion morphology were strong correlates of ISR [71]. Small vessel treatment is a challenging setting because of an increased risk of restenosis and the need for repeat revascularization [92]. Contemporary newer-generation DES significantly reduced both late lumen loss and restenosis in these complex lesions [93]. The use of drug-coated balloons (DCB) instead of DES for the treatment of native small vessel CAD has emerged as a valuable alternative strategy. In a recent meta-analysis that included 5 randomized trials and 1459 patients, the use of DCB as compared to DES was associated with similar rates of TLR and restenosis, while a lower risk of ST was observed [94]. Severe calcified lesions are strongly associated with higher rates of ISR [95]. Coronary calcium may cause poor drug delivery, inefficient stent expansion and wall apposition, and even polymer disruption [95]. To achieve ideal stent apposition and long-term patency, adequate lesion preparation using balloon-based procedures, atheroablative devices, and intravascular lithotripsy is crucial [96]. The pattern of restenosis is another important predictor of ISR. Specifically, diffuse ISR was associated with significantly higher TLR rates as compared with focal pattern in a study that included 392 patients with 481 de novo DES-ISR [97]. Coronary bifurcation PCI is associated with higher rates of restenosis and TLR as compared with non-bifurcation lesions, and the implemented stenting technique can substantially affect clinical outcomes [98,99]. Aorto-ostial lesions are composed mainly of sclerotic and highly tenacious plaques, are often eccentric, and have a high plaque burden. For these lesions, restenosis rates have been reported to be higher as compared to stented non-ostial lesions [100].

Stent type, stent strut thickness, stent length, and stent diameter have been related to the risk of ISR. In the largest analysis investigating ISR thus far published, the use of first-generation DES versus BMS and second-generation DES versus first-generation DES were independent predictors of lower rates of restenosis [101]. Stent struts thickness, affecting the extent of arterial wall injury at the time of implantation and local blood rheology after implantation, impacts the inflammatory response strength at the target lesion, re-endothelization process, struts coverage, and neointima formation [102]. Thinner struts have been associated with lower inflammation at the stented arterial segment, with less thrombogenicity and less neointimal hyperplasia and a lower risk of clinically-driven TLR and target vessel revascularization [12,102,103]. Stent length represents another stent-related factor associated with higher rates of stent failure, either ST or ISR. In the GRAND-DES (Grand Drug-Eluting Stent) registry, which includes 8035 patients undergoing stenting for a single lesion with newer-generation DES, stents longer than 40 mm were associated with a higher rate of TLR and early ST at a median follow-up of 730 days [104].

Stent underexpansion and stent struts malapposition have been previously defined and have been associated with an increased risk of ISR as well as ST. Additionally, there is evidence that there is a higher risk of ISR and TLR when there is a stent gap, which is characterized as a discontinuous coverage of a coronary lesion between two stents [105]. Local drug distribution is hampered by DES fracture, and the metallic platform support is compromised. Right coronary artery stenting, severe vascular tortuosity or angulation, and lengthier or overlapping stents are known risk factors for stent fractures. On the other hand, stents having a greater diameter or an open-cell construction seem to be less likely to fracture. The need for revascularization of fractured stents ranges from 15% to 60%, while the incidence of DES fracture has been reported to be between 1% and 8%.

Available intracoronary imaging modalities allow a detailed characterization of the underlying ISR substrate and the selection of the most appropriate treatment strategy. For instance, in 2019, a new OCT classification system to characterize the most relevant mechanism of ISR and to guide treatment was proposed [106]. This classification includes 5 types of ISR: mechanical or type I, further classified as Ia (under expansion) and Ib (stent fracture); biological or type II which includes type IIa (neointimal hyperplasia), type IIb (neoatherosclerosis without calcifications), and type IIc (neoatherosclerosis with calcifications); mixed-causes or type III; chronic total occlusion or type IV; and 2-layer or type V ISR. Imaging characterization of the underlying mechanism is of crucial importance to guide the selection of the treatment strategy [27]. It is extremely important to identify the cause of ISR (DES under expansion vs. neointimal hyperplasia/neoatherosclerosis), differentiate between 1-layer and 2-layer ISR, and distinguish ISR as focal, diffuse, or occlusive phenotype [107]. All these aspects are essential to select the most appropriate treatment modality.

## 5. Conclusions

Iterations in stent technologies, improvements in PCI techniques, and advances in pharmacotherapy have markedly reduced the risk of ST and ISR. However, given the number of PCIs performed every year around the world, the global burden of ISR and ST represents a public health issue even with contemporary DES platforms. The understanding of mechanisms and risk factors for these two PCI complications has been of fundamental importance for the parallel evolution of stent technologies. Because the pathogenesis of ST and ISR is multifactorial, differentiated risk stratification is needed. The identification of patient-, stent-, lesion-, and procedure-related predictors is particularly important, as preventing ST and ISR is the most efficient way to combat them.

## Figures and Tables

**Table 1:** Main features of durable-polymer drug-eluting stents.

	Endeavor	Xience	Promus	Resolute
**Platform material**	CoCr	CoCr	PtCr	CoCr
**Strut thickness (μm)**	91	81	81	91
**Polymer material**	MPC/LMA/HPMA/3-MPMA	PBMA/PVDF-HFP	PBMA/PVDF-HFP	PBMA/PHMA/PVP/PVA
**Coating** **distribution**	Circumferential	Circumferential	Circumferential	Circumferential
**Polymer thickness (μm)**	4.8	8	8	4.8
**Drug released**	Zotarolimus	Everolimus	Everolimus	Zotarolimus

CoCr: cobalt chromium; HPMA: hydroxypropyl methacrylate; LMA: lauryl methacrylate; MPC: methacryloyloxyethyl phosphorylcholine; PBMA: poly n-butyl methacrylate; PHMA: polyhexyl methacrylate; PtCr: platinum chromium; PVA: polyvinyl acetate; PVDF-HFP: co-polymer of vinylidene fluoride and hexafluoropropylene; PVP: polyvinyl pyrrolidinone; 3-MPMA: trimethoxysilylpropyl methacrylate.

**Table 2:** Main features of biodegradable polymer drug-eluting stents.

	Synergy	Synergy Megatron	BioMatrix/Nobori	Ultimaster	COMBO	Orsiro	MiStent	BioMime	Supraflex	Yukon Choice PC	Firehawk
**Platform material**	PtCr	PtCr	Stainless steel	CoCr	Stainless steel	CoCr	CoCr	CoCr	CoCr	Stainless steel	CoCr
**Strut thickness (μm)**	74	89	120	80	100	60	64	65	60	87	86
**Polymer material**	PLGA	PLGA	PDLLA	PDLLA-PCL	PDLLA/PLGA	PLLA	PLGA	PLLA/PLGA	PLLA/PLCL/PVP	PDLLA	PDLLA
**Coating distribution**	Abluminal	Abluminal	Abluminal	Abluminal	Abluminal	Circumferential	Circumferential	Circumferential	Circumferential	Abluminal	Abluminal
**Polymer thickness (μm)**	4		10	15	5	7	15	2	4–5	5	10
**Drug released**	Everolimus	Everolimus	Biolimus A9	Sirolimus	Sirolimus	Sirolimus	Sirolimus	Sirolimus	Sirolimus	Sirolimus	Sirolimus
**Additional features**					Circumferential coating of anti-CD34 antibodies	Silicon carbide additional coating				Microporous PEARL surface for better endothelial cell adhesion	

CoCr: cobalt chromium; PCL: poly-Caprolactone; PDLLA: poly-D, L-lactic acid; PLCL: poly-L-lactide-co-Caprolactone; PLGA: poly-lactic co-glycolic acid; PLLA: poly-L-lactic acid; PtCr: platinum chromium; PVP: polyvinyl pyrrolidone.

**Table 3:** Main features of polymer-free drug-eluting stents.

	BioFreedom Ultra	Cre8	Coroflex ISAR NEO
**Platform material**	CoCr	CoCr	CoCr
**Strut thickness (μm)**	84	70–80	55–65
**Drug released**	Biolimus A9	Amphilimus	Sirolimus
**Surface modification technique**	Abluminal microporous surface coating	Abluminal Reservoir-based coating	Abluminal microporous surface coating
**Additional features**		BioInducer surface (<0.3 μm) covalently bonded to the CoCr platform to limit risk of allergic reaction and platelet adhesion	Probucol as matrix-builder and is a highly lipophilic, lipid-lowering agent with antioxidant effects

CoCr: cobalt chromium.

**Table 4:** Main features of bioresorbable scaffolds.

Device	Backbone	Coating	Strut Thickness (μm)	Eluted Drug	Bioresorption Time (Months)
**Bioresorbable polymer**					
**Absorb BVS**	PLLA	PDLLA	157	Everolimus	24–48
**DESolve Nx**	PLLA	Polylactide-based	150	Novolimus	24
**DESolve Cx**	PLLA	Polylactide-based	120	Novolimus	24
**Fantom**	DAT-PC	DAT-PC	125	Sirolimus	36
**Bioresorbable metal**					
**DREAMS 1G**	Magnesium alloy	PLGA	125	Paclitaxel	9–12
**Magmaris**	Magnesium alloy	PLLA	150	Sirolimus	9–12

DAT-PC: desaminotyrosine polycarbonate; PLLA: poly-L-lactic acid; PDLLA: poly-D, L-lactic acid; PLGA: poly-lactic co-glycolic acid.

## Data Availability

Not applicable.
